# Temperature-Responsive Biocompatible Copolymers Incorporating Hyperbranched Polyglycerols for Adjustable Functionality

**DOI:** 10.3390/jfb2030173

**Published:** 2011-08-23

**Authors:** Darlene K. Taylor, Friederike L. Jayes, Alan J. House, Melony A. Ochieng

**Affiliations:** 1 Department of Chemistry, North Carolina Central University, Durham, NC 27707, USA; E-Mail: mochieng@eagles.nccu.edu; 2 Center for Uterine Fibroid Biology and Therapy, Department of Obstetrics and Gynecology, Duke University, Durham, NC 27710, USA; E-Mail: friederike.jayes@duke.edu; 3 Department of Pharmaceutical Sciences, BRITE, North Carolina Central University, Durham, NC 27707, USA; E-Mail: ajhouse@nccu.edu

**Keywords:** drug delivery, hyperbranched polyglycerol, temperature responsive, cell biocompatibility

## Abstract

Temperature-triggered copolymers are proposed for a number of bio-applications but there is no ideal material platform, especially for injectable drug delivery. Options are needed for degradable biomaterials that not only respond to temperature but also easily accommodate linkage of active molecules. A first step toward realizing this goal is the design and synthesis of the novel materials reported herein. A multifunctional macromer, methacrylated hyperbranched polyglycerol (HPG-MA) with an average of one acrylate unit per copolymer, was synthesized and copolymerized with N-isopropylacrylamide (NIPAAm), hydroxyethyl methacrylate-polylactide (HEMAPLA) and acrylic acid (AAc). The potential to fully exploit the copolymers by modification of the multiple HPG hydroxyl groups will not be discussed here. Instead, this report focuses on the thermoresponsive, biocompatible, and degradation properties of the material. Poly(NIPAAm-co-HEMAPLA-co-AAc-co-HPG-MA) displayed increasing lower critical solution temperatures (LCST) as the HPG content increased over a range of macromer ratios. For the copolymer with the maximum HPG incorporation (17%), the LCST was ∼30 °C. In addition, this sample showed no toxicity when human uterine fibroid cells were co-cultured with the copolymer for up to 72 h. This copolymer lost approximately 92% of its mass after 17 hours at 37 °C. Thus, the reported biomaterials offer attractive properties for the design of drug delivery systems where orthogonally triggered mechanisms of therapeutic release in relatively short time periods would be attractive.

## 1. Introduction

A number of synthetic hydrogels with a lower critical solution temperature (LCST) below body temperature have been touted as promising injectable drug delivery systems [1,2,3,4,5,6,7]. Such biomaterials swell in water and typically undergo a phase transition to gel immediately after reaching their LCST. Viable representatives of these thermally smart polymers include polysaccharide derivatives [8], poly(N-isopropylacrylamide) (PNIPAAm) [9,10,11], and poly(ethylene glycol) [6,8]. However, all these examples represent hydrophilic materials that are biologically non-degradable on any useful timescale. Biodegradable macromers such as hydrophobic lactides are often copolymerized with thermogelling polymers to facilitate bioadsorption and clearance from the body at physiological temperatures [12]. PNIPAAm-based hydrogels incorporating poly(lactic acid) (PLA) macromers are routinely investigated as injectable bulking biomaterials since the ester linkages of PLA are hydrolytically degraded in the presence of water and the LCST can be tuned by the monomer feed ratio. Further improvements to the hydrogel delivery system are realized by copolymerizing small amounts of hydrophilic molecules, such as acrylic acid, to enhance the bioadsorption of the hydrolytically degraded copolymer [13].

Although the copolymers discussed above represent feasible options for developing *in situ* gelling biomaterials—indeed a prototype PNIPAAm–based delivery system has been valuable in animal models with compromised ventricular architecture of the heart [9]—limitations exist with respect to extending the utility of one delivery system to more than one application. No ideal drug delivery system has been designed to date. Molecularly engineered materials that work by mechanistically and kinetically independent delivery techniques would, however, be transformative to the field. Options are needed for degradable biomaterials that not only respond to temperature but also easily accommodate chemical linkage of active molecules. Such a platform could potentially utilize orthogonally triggered mechanisms (such as temperature stimulated entrapment and pH programmed linkage) to provide targeted and controlled delivery of therapeutic agents.

This study describes the synthesis of injectable PNIPAAm-based thermogelling copolymers that incorporate hyperbranched polyglycerol (HPG) macromers for chemically modifiable functional sites. We envision this design yielding a novel environmentally responsive copolymer that not only responds to programmed stimuli but also incorporates copious terminal hydroxyl groups for continued advancement of this delivery platform. We chose HPG macromers to impart functionality to our copolymers because of their internal cavities suitable for small molecule interaction, large number of modifiable surface hydroxyl groups, and excellent biocompatibility [14,15,16,17,18,19,20]. A convenient pathway to well-defined HPGs was recently reported by Sunder *et al.* based on the anionic polymerization of a latent AB_2_-type glycidol monomer using ring-opening multibranching polymerization (ROMBP) [21]. Slow addition of the monomer permits its exclusive reactivity with the growing multifunctional hyperbranched polymer, leading to well-defined growth of the macromolecules in a ‘living’ type of polymerization. A rapid proton exchange equilibrium maintains all hydroxyl groups present as potentially active propagation sites, thus leading to random, but controlled, branching. Other polymer approaches [22,23] cannot easily provide these properties without significant increases in the number of synthetic steps and the cost of synthesis. Thus, HPGs may now be obtained in a single step with properties that rival the very popular dendritic materials platform [24]. Kojima *et al.* have reported HPGs with all available terminal hydroxyl groups modified by succinylation and further altered with various oligo(ethylene glycol) monoethers [25]. These materials are temperature-sensitive, but represent significantly different materials from those reported here. Our goal was to develop a temperature-responsive domain with mostly unaltered hydroxyl groups available for additional modification of moieties that are orthogonally exploited by a trigger other than temperature. To our knowledge, noone has yet described the preparation of biodegradable and thermoresponsive copolymers covalently linked with HPG macromers that can be further manipulated by an orthogonal trigger.

**Scheme 1:**
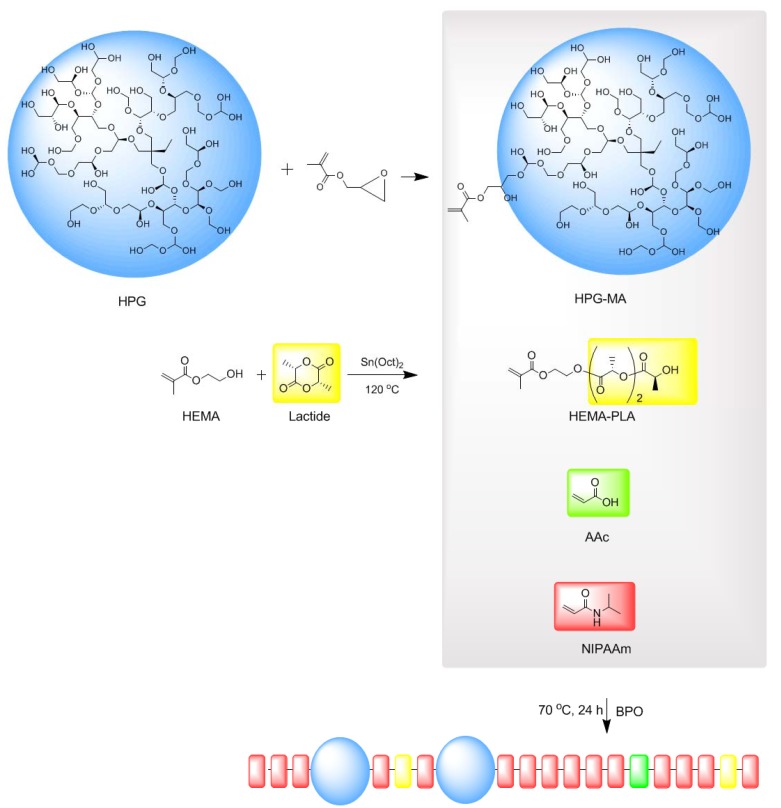
Thermogelling Biomaterials from the Acrylic Macromers Methacryalted-Hyperbranched Polyglycerol (HPG-MA) ^a^ and 2-Hydroxyethyl Methacrylate-poly(lactic acid) (HEMAPLA) ^b^ Copolymerized with Monomers N-Isopropylacrylamide (NIPAAm) and Acrylic Acid (AAc) by Radical Polymerization. ^a^ Methacrylate moieties that enable incorporation of HPG into the copolymer are introduced in the first step. The schematic illustration of this reaction is simplified, recognizing that on average one out of 29 pendant HPG hydroxyl groups reacted in the methylation step; ^b^ The HEMAPLA macromer is prepared as a standalone reaction. The resulting four component copolymer is a branched statistical copolymer.

A free radical polymerization strategy is described to obtain the four component functional copolymer. Each component plays a specific role in the resulting copolymer function: *N*-isopropylacrylamide provides thermogelling with a LCST below physiological conditions, poly(lactic acid) (PLA) provides biodegradability through hydrolytic bond cleavage, acrylic acid (AAc) provides a hydrophilic component to increase the transition temperature of the copolymer after hydrolysis, and HPG provides a number of hydroxyl groups available for chemical modification to covalently attach fluorescent tags for biomarkers or pH triggered linkers terminated with bioactive molecules. The PLA macromer is incorporated as side components linked to 2-hydroxyethyl methacrylate (HEMA), yielding HEMAPLA. HEMA is easily coupled to PLA and renders an olefin group that can be copolymerized with the other acrylic macromers; thus HEMAPLA was chosen over PLA alone to facilitate chemical synthesis. Similarly, HPG was functionalized with methacrylate groups, HPG-MA, in order to realize its incorporation in the copolymer. Thus, this study presents a method to yield a series of copolymers with different ratios of NIPAAm, HEMAPLA, AAc, and HPG-MA. This report does not focus on demonstrating the various substitutions possible at the hydroxyl region of the HPG containing copolymer. Instead, we take the first step in exploiting this material by focusing on the synthesis of the copolymer (shown in Scheme 1) and testing its thermoresponsive, biocompatible, and degradation properties. The copolymers are characterized by nuclear magnetic resonance (NMR) spectroscopy and gel permeation chromatography (GPC). Solutions of the macromers were characterized for their phase-transition properties by differential scanning calorimetry (DSC) and optical absorption. Candidate copolymers were analyzed by mass spectrometry, and cytocompatibility and degradation properties were also assessed.

## 2. Experimental Section

### 2.1. Materials

HPG (M_n,MALDI_ = 1096 g mol^−1^, M_w_/M_n_ = 1.13) was prepared according to the literature by controlled anionic polymerization of glycidol [21], and the detailed characterization is reported in the supplementary information. The average number of terminal hydroxyl groups per HPG molecule was approximately 29 as determined by the relative integrals from the inverse gated ^13^C NMR spectra. Glycidyl methacrylate (GMA), acrylic acid (AAc), and N-isopropylacrylamide (NIPAAm) were purchased from Sigma-Aldrich (St. Louis, MO). AAc was purified immediately prior to use by passage through a basic alumina column. NIPAAm was recrystallized from hexane and vacuum dried. Benzoyl peroxide (BPO), stannous 2-ethylhexanoate [(Sn(Oct)_2_],(3S)-cis-3,6 dimethyl-1,4-dioxane,-2,5 dione (98%) (L-lactide), 4-(N,N-dimethylamino)pyridine (DMAP), anhydrous dimethyl sulfoxide (DMSO), anhydrous 1,4-dioxane, methyl sulfoxide-d_6_ (99.9% atom D), anhydrous methanol, tetrahydrofuran (THF), and phosphate-buffered saline (PBS) were purchased from Fisher Scientific (Pittsburgh, PA). All polymerizations were carried out under a dry nitrogen atmosphere.

### 2.2. Synthesis

#### Synthesis of HPG-MA

Methacrylated HPG was synthesized essentially as described by Oudshoorn *et al.* [26]. As shown in Scheme 1, HPG-MA structures were prepared by functionalization of the HPG hydroxyl groups with glycidyl methacrylate.

#### Synthesis of HEMAPLA

HEMAPLA was synthesized by ring-opening polymerization of L-lactide initiated by HEMA with Sn(Oct)_2_ as a catalyst (Scheme 1). Equivalent molar ratios of HEMA and lactide were reacted at 110 °C in a nitrogen atmosphere for 1 h in the presence of catalyst Sn(Oct)_2_ (121.5 mg, 1 mol% with respect to HEMA). The cooled reaction mixture was dissolved in THF and precipitated in ice cold water. The precipitate was dissolved in ethyl acetate and filtered to remove the remaining solids. The filtrate was dried over MgSO_4_ and concentrated under reduced pressure to obtain purified HEMAPLA.

#### Synthesis of Poly(NIPAAm-co-HEMAPLA-co-AAc-co-HPG-MA)

Poly(NIPAAm-co-HEMAPLA-co-AAc-co-HPG-MA) copolymers were synthesized by free radical polymerization (Scheme 1). All glassware was dried at 120 °C for 12 h and flamed in a vacuum to eliminate moisture before use. A 5 wt% solution of monomers (NIPAAm and AAc) and macromers (HEMAPLA and HPG-MA) in 1,4-dioxane was introduced in a dry, preweighted round-bottom flask equipped with rubber septum and a magnetic stir bar. A solution of BPO (7.2 × 10^−3^ mol/mol monomer) in 1,4-dioxane was added. The polymerization was conducted at 70 °C for 24 h under nitrogen atmosphere. The copolymer was purified by precipitation in hexane followed by precipitation from THF into diethyl ether and vacuum dried.

### 2.3. Characterization

#### Nuclear Magnetic Resonance

^1^H and ^13^C NMR spectra were recorded in deuterated dimethyl sulfoxide (unless otherwise noted) on a Varian spectrometer operating at 500 MHz. Chemical shifts (δ) are reported in parts per million (ppm) relative to tetramethylsilane (TMS 0.0 ppm (^1^H) and 77.0 ppm (^13^C)).

#### Matrix-Assisted Laser Desorption and Ionization Time-of-Flight Mass Spectrometry (MALDI-TOF-MS)

An Applied Biosystems Voyager-DE PROmass spectrometer equipped with a nitrogen laser (337 nm) was used to collect mass spectra data. A 32 ns delay was applied before ions were accelerated to 25 kV and positive ions detected. Additionally, the grid and guide wire voltages were set at 90% and 0.15% of the applied acceleration voltage, respectively, to focus the beam of ions. Typically, 40 laser shots were averaged for each spectrum. 4′-hydroxyazobenzene-2-carboxylicacid (HABA) was used as the matrix. The 1-100 mM matrix and analyte stock solutions were prepared as methanol solutions and were mixed in microcentrifuge tubes at matrix/analyte ratios varying from 1:1 to 1000:1; 1–2 μL of this solution was applied to the sample plate and air-dried.

#### Electrospray Ionization Time-of-Flight Mass Spectrometry (ESI-TOF MS)

ESI-TOF mass spectrometry was performed using a Micromass Q-tof *micro* (Waters Corp., Milford, MA). Samples were dissolved in methanol (0.1 or 1 mg mL^−1^, HPG or HPG-MA, respectively) and passed (0.5–1 μL min^−1^) through a nano-ESI source operated in positive ion mode with a capillary voltage of 2–3 kV, sample cone voltage of 33 V, source temperature of 90 °C and desolvation temperature set at 180 °C. Nitrogen was used as the nebulizing gas. Sodium iodide cesium iodide was used to calibrate masses from *m/z* 400 to 1990 Da. Data was collected in continuum mode for 3–10 min over the same mass range with a 1 s scan time and 0.1 s inter scan time. Spectra were collected and processed using Masslynx 4.0 software (Waters).

#### Gel Permeation Chromatography (GPC)

The molecular weights and molecular weight distributions of synthesized copolymers were determined by GPC unless otherwise noted. A Waters Alliance System, Waters 2695 Separations Module and Waters 2414 Refractive Index Detector (Waters Associates Inc., Milford, MA) were utilized. Approximately 20–30 mg of copolymer was dissolved in THF and the GPC analysis was performed at 35 °C. The flow rate was 1.0 mL/min. A polystyrene standard kit was used for molecular weight elution volume calibration.

#### Differential Scanning Calorimetry (DSC)

Measurements were carried out on a Perkin-Elmer Pyris 1 DSC equipped with a cyrofill liquid nitrogen cooling system. LCSTs of the copolymer solutions in PBS (16.7 wt%) were studied using a scanning rate of 5 °C/min over a temperature range of −10 to 45 °C. The temperature at the maximum of the endothermal peak was recorded as the LCST [27].

#### UV-Vis

LCSTs of the copolymer solutions in PBS (16.7 wt%) were studied by measuring optical absorption. A SpectraMax M5e Microplate Reader (Molecular Devices, Inc., Sunnyvale, CA) was operated in single wavelength mode at 500 nm over a temperature range of 25 to 45 °C. The LCST of each copolymer was determined in triplicate.

#### In vitro degradation

The cold copolymer solutions in PBS (16.7 wt%) were poured into 2 mL vials and incubated for different periods of time at 37 °C. At predetermined times, samples were quenched in liquid nitrogen and frozen until needed for further studies. The frozen samples were lyophilized and the molecular weights of the copolymers were determined by GPC.

### 2.4. Cytotoxicity Assay

Sterile Dulbecco's modified Eagle's medium (DMEM-F12) was purchased from Lonza (Walkersville, MD). FBS (fetal bovine serum) and antibiotics were obtained from Sigma. Human uterine fibroid tissue was obtained from the existing IRB approved infrastructure of the Uterine Fibroid Tissue Repository which is part of Duke University School of Medicine Research Foundation's tissue banking operation. Uterine fibroids present a significant health problem to women in that, by age 50, 70–80% of all women are affected and no reliable medical treatments exist, short of hysterectomy. We envision the copolymers serving as a potential protection for local delivery of anti-fibroitic drugs. The fibroid cells were isolated by enzymatic digestion of fibroid tissue obtained at hysterectomy and cultured in DMEM-F12 medium supplemented with antibiotics, antimycotic and 10% FBS. In general, third passage cells were used in the cytotoxicity studies. Polymer solutions (16.5 wt% in PBS) were filtered through 0.22 μm filters (VWR 28145-501 polyethersulfone sterile filters). Cells were plated in 24-well plates and incubated for 24–48 h until 80% confluent. Then, cells were washed with prewarmed PBS and incubated with fresh media and HPG containing copolymer hydrogel or HPG macromer (0–4.8 mg/mL) for 72 h. Each concentration was measured four times. Cytotoxicity was assessed with a methyl tetrazolium salt (MTS) assay kit (Promega, CellTiter96^®^AQ_ueous_ Non-Radioactive Cell Proliferation Assay) following the protocol provided by the manufacturer and a 3 h incubation time. Results are expressed as percent viability relative to control cells grown in media alone (100% viability). The assay was repeated with fibroid cells from a different patient. Microscopy was used to help verify assay results.

## 3. Results and Discussion

### 3.1. Synthesis of HPG-MA

**Figure 1:**
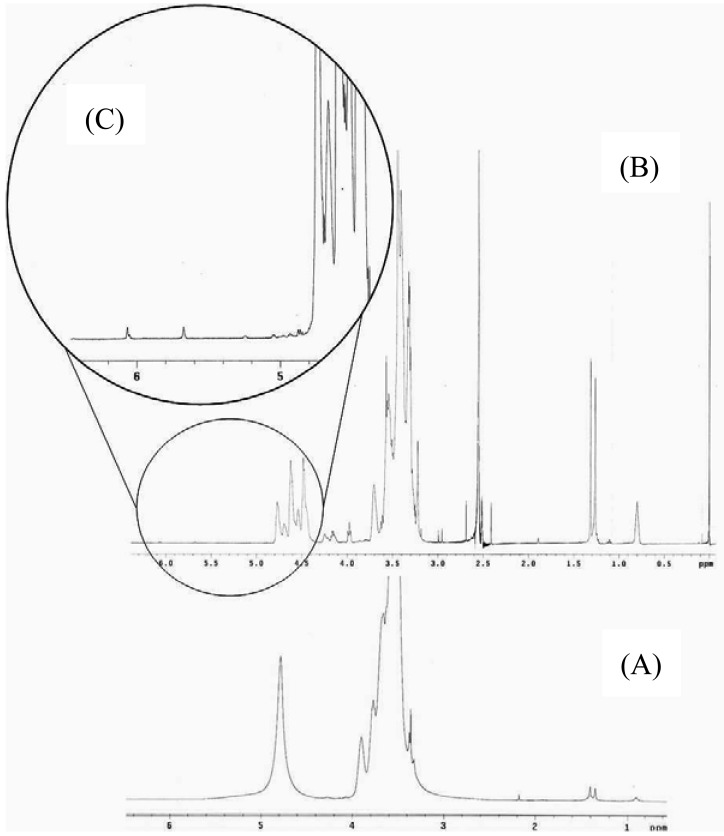
^1^H NMR spectra (CD_3_OD) of hyperbranched polyglycerol (HPG) obtained from anionic polymerization initiated with 1,1,1-tris(hydroxymethyl)propane (**A**). ^1^H NMR spectra (DMSO-d_6_) of methacrylated HPG (DS = 0.16) (**B**); the insert shows the magnified region where the acylate peaks of HPGMA appear (**C**).

The multifunctional, degradable thermoresponsive copolymer used in this study was synthesized in three steps as depicted in Scheme 1. The first reaction depicted in this scheme corresponds to the method used to prepare methacrylated HPG macromer. The methacryloyl group was directly linked to the starting HPG by transesterification. ^1^H NMR analysis of the product was consistent with results reported by Oudshoorn *et al.* [26]. In addition to the four methylene and one methine (broad multiplet at 3.4 ppm) and one hydroxyl proton (4.8 ppm) originating from the monomer repeat units of HPG, new peaks were detected. ^1^H NMR spectra shown in Figure 1 confirmed the incorporation of the ethacryloyl group with the observation of methyl (1.8 ppm) and acrylate protons (5.67 and 6.08 ppm, shown more clearly with the enlarged insert). Integration of the peak areas for these acrylate protons relative to the HPG [terminal hydroxyl] protons suggests that the fraction of HPG macromer derivatized with methacrylate groups to yield HPG-MA was 0.16. This low degree of conversion implies that the HPG is mostly unaltered but when substitution does occur, on average ∼one out of 29 hydroxyl groups of HPG were substituted with a methacryloyl group.

**Figure 2:**
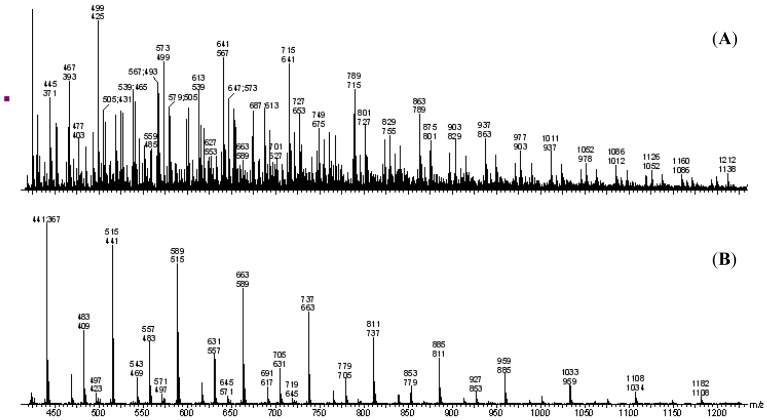
ESI-TOF of (**A**) HPG-MA and (**B**) HPG macromer precursor.

MALDI-TOF was used to determine the mass of HPG-MA (M_n_ = 1,253 g/mol, M_w_/M_n_ = 1.13, data not shown). The MALDI MS of HPG-MA was consistent with the ion fragmentation pattern obtained by ESI MS which is shown in Figure 2A. The top mass number in the figure corresponds to the peak *m/*z value, with a charge (*z*) of +1 evident in all labeled peaks (data not shown). The bottom mass number represents a delta mass of 74 from the peak *m/*z value, corresponding precisely to the repeat unit mass of the C_3_H_6_O_2_ interval (glycidol, MW = 74). There are two significant series of peaks that differ by mass unit 74 within their respective series (*m/z* = …499, 573, 647, 721… and *m/z* =…641, 715, 789, 863…). The mass difference between the two series (*i.e.*, 641 minus 499 or 715 minus 573, *etc.*) equals 142, corresponding precisely to the mass of one methacrylated glycidol unit (MW = 142). These results suggest that only one methacryloyl group is incorporated into HPG-MA, consistent with data collected using ^1^H NMR (Figure 1).

Observing two major ion series is consistent with the expected polydispersed copolymer population. For instance, because most of the polymer chains in HPG share the same initiator (1,1,1-tris(hydroxymethyl)propane), the mass distribution among dispersed macromers would vary by a multiple of 74 dependent on the number of glycidol monomers. Thus, mass spectrum analyses of the dispersed macromers would yield superimposed peaks that vary by *m/z* = 74 following the loss of glycidol monomers during ESI. The only variation to superimposition detected resulted from the loss of one mass of 142, instead of 74, suggesting some copolymer fragments contain one methacrylated glycidol while others do not. No evidence of multiple methacryloyl incorporation was observed.

For comparison, Figure 2B shows the precursor HPG macromer with three major series of molecular weight distributions having a repeat unit mass m/z = 74 within the series (*m/z* = 499, 573, 647…; *m/z* = 527, 601, 675…; and *m/z* = 559, 633, 707…). The difference between these series may correspond to incorporation of a cyclic derivative of glycidol as previously reported [21]. No peaks are observed in HPG with a mass delta of 142. Together, this data confirms that glycidyl methacrylate was incorporated into the hyperbranched structure. No evidence of multiple methacryloyl incorporation was observed. [This supports ^1^H-NMR data discussed above, which implied that on average ∼one out of 29 hydroxyl groups of HPG are substituted with a methacryloyl group.]

### 3.2. Synthesis of HEMAPLA

**Figure 3:**
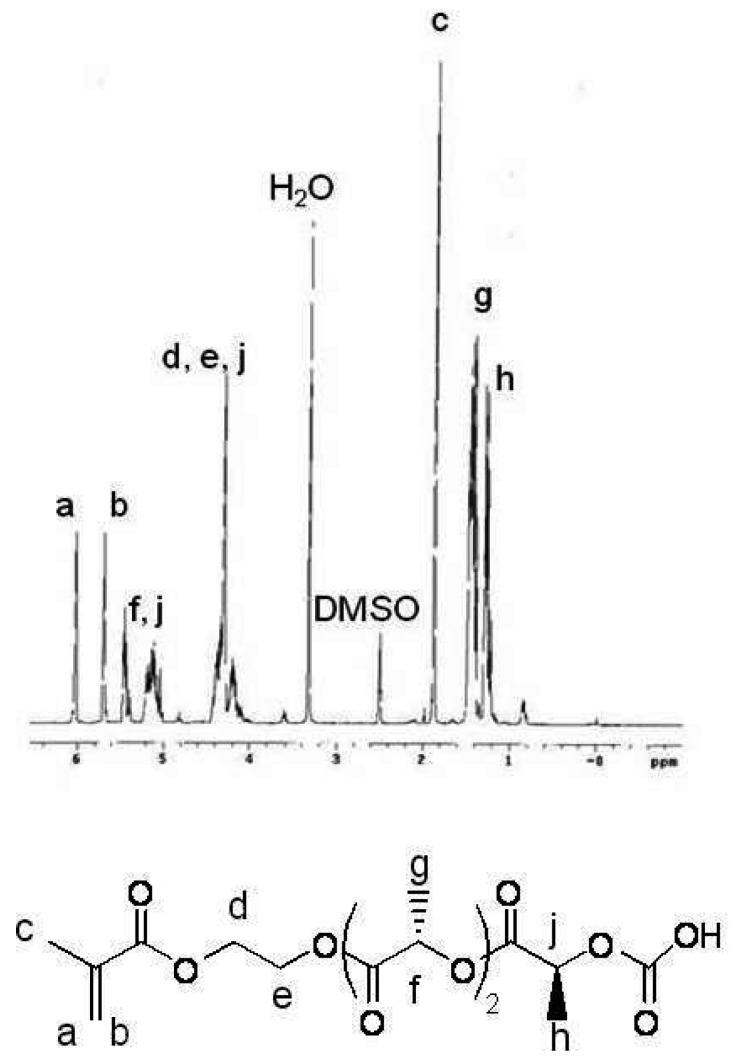
^1^H NMR spectra (DMSO-d_6_) of HEMAPLA.

Prior to the copolymer polymerization, the macromer HEMAPLA was prepared and its synthesis confirmed by ^1^H NMR (Figure 3). The proton peaks are in agreement with the molecular structure of HEMAPLA. The number average length of PLA units per macromer was determined from the ^1^H NMR spectrum by calculation from the ratio of the integrals of hydrogen peaks from PLA (peaks c, f, j, and h) relative to the double bond hydrogen peaks (peaks b and a at 5.6 and 6.1 ppm, respectively). A PLA repeat unit of 3 was determined and found to be in agreement with the molar feed ratio of HEMA to L-lactide (1:1) utilized in the synthesis of HEMAPLA.

### 3.3. Synthesis of Copolymers

A series of copolymers with different relative molar amounts of HPG-MA were prepared by free radical polymerization. The low degree of methacrylate substituted hydroxyl groups (an average of one out of 29 groups) ensured that on average only one link occurred between the polymer backbone and the incorporated HPG macromer. This degree of substitution also minimizes the probability of HPG initiated crosslinks in the final copolymer products. Any HPG-MA not incorporated into the co-polymer was isolated and removed during the workup. The four component copolymers were synthesized with different monomer and macromer feed ratios and their properties are summarized in Table 1.

**Table 1:** Characteristics of poly(NIPAAm-co-HEMAPLA-co-AAc-co-HPG-MA) copolymers with different HPG feed ratios.

**(NIPAAm∣HEMAPLA∣AAc∣HPG-MA)**												
										**GPC**		**LCST (^°^C)^a^**
**Sample ID**	**Yield**	**Feed Ratio**				**^1^H NMR (M_n_)**				**M_n_**	$\frac{M_{w}}{M_{n}}$	
HPG High	88%	(80∣	10∣	1∣	9)	(79∣	1∣	3.3∣	17)	3689	1.7	28 ± 0.1
HPG Med	83%	(86∣	7∣	1∣	6)	(85∣	8∣	0.8∣	6)	3455	1.7	24 ± 0.2
HPG Low	87%	(85∣	10∣	1∣	4)	(85∣	11∣	0.2∣	4)	4465	1.5	22 ± 0.1
Control	90%	(87∣	10∣	3∣	0)	(86∣	12∣	1.8∣	0)	1253	1.5	20 ± 0.4

a 16.7 wt% in PBS, measured by DSC.

**Figure 4:**
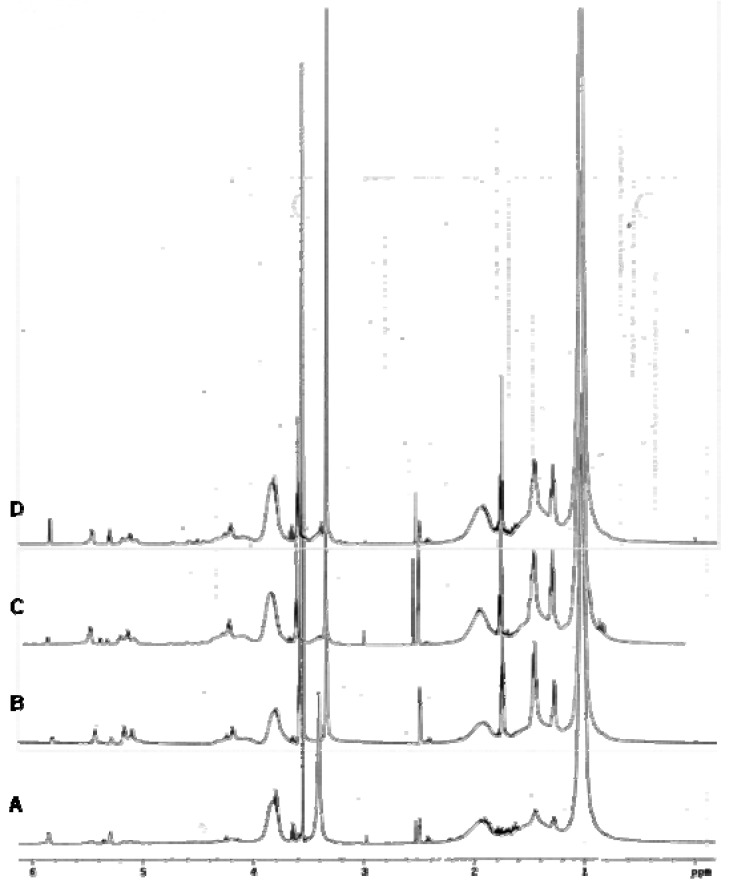
(**a**) ^1^H NMR spectra (DMSO-d_6_) of copolymers of poly(NIPAAm-co-HEMAPLA-co-AAc-co-HPG-MA), where the spectra represent (**A**) *Control*; (**B**) *HPG Low*; (**C**) *HPG Med*; and (**D**) *HPG High*.

The obtained ^1^H NMR data verified that the polymer building blocks (four acrylate groups of NIPAAm, HEMAPLA, AAc, and HPG-MA respectively) reacted at the intended molar feed ratios. Figure 4 shows the stacked ^1^H NMR spectra for all the copolymers synthesized. Proton peaks characteristic of the monomer NIPAAm (methyl, 1.04 ppm) or macromers HEMAPLA (where the PLA component was observed at 5.1 ppm) and HPG-MA (methylene and methine, 3.4 ppm) were observed. The existence of AAc (-COOH) units and their relative amounts was approximated from the visible and integratable peak at 11.7 ppm. This approach was taken instead of the more common and accurate titration approach [28,29] because of the presence of hydroxyl groups on both the HPG and in the side chain acid groups of AAc.

**Figure 5:**
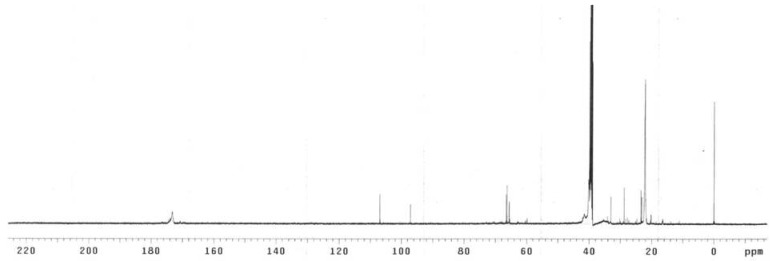
^13^C NMR spectra for poly(NIPAAm-co-HEMAPLA-co-AAc-co-HPG-MA) where the spectrum represents polymer sample *HPG High*.

We again used ^1^H NMR spectroscopy to confirm the incorporation of HPG macromer into the copolymer. Notice in Figure 4: (A) the *Control* copolymer which was prepared with no HPG-MA feed, (B) the *HPG Low* copolymer, (C) the *HPG Med* copolymer, and (D) *the HPG High* copolymer. A singlet peak is observed at δ = 3.4 ppm in Figure 4A arising from water in DMSO-d_6_. This peak is shifted to ∼3.3 ppm in Figure 4B–D, and in its place a small broad multiplet peak emerges at δ = 3.4 ppm (CH, CH_2_ protons of HPG). Furthermore, this broad peak occurring at 3.4 ppm grows in intensity (*i.e.*, increased integral area) in going from spectra shown in Figure 4B to 4D. Peaks corresponding to the polymerization solvent, 1,4-dioxane, also shift to this region, but ^13^C spectra of the *HPG High* sample shown in Figure 5 confirmed that little solvent remained as the corresponding peak at ∼67 ppm was barely detected above the baseline noise (number of scans = 10,000). Thus, in calculating the HPG content in each copolymer, the water signal was negated by comparing the relative ratio of H_2_O: DMSO signal (2.5 ppm) in the control sample to the H_2_O:DMSO in each of the respective copolymers. Using this approach, the signal at 3.4 ppm more closely reflects the approximate HPG content. The monomer compositions in the copolymers were found to be similar to the feed ratios as shown in Table 1.

The molecular weights of the poly(NIPAAm-co-HEMAPLA-co-AAc-co-HPG-MA) copolymers were determined by GPC. The molecular weights obtained for the synthesized copolymers were low due to the monomer to initiator feed ratio. The molecular weight decreases as the HPG-MA feed ratio content increases. This result may be a result of steric hindrance as it is more difficult to easily incorporate the bulky HPG group into the polymer backbone via the approximately one acrylate group per HPG molecule. All of the copolymers have molecular weights between 1,200 and 3,700 g mol^−1^, and a polydispersity index of 1.5–1.7. Considering the fact that the GPC column was calibrated with linear polystyrene, the measured M_n_ values for *HPG High*, *HPG Med* and *HPG Low* are expected to be problematic as the hyperbranched structure of the HPG component does not accurately correspond to the linear polystyrene calibrant. However, the GPC-determined molecular weight distribution of the copolymers can be used as a reference.

Further analysis of the molecular weight and its distribution for the *HPG High* and *Control* samples was obtained from MALDI MS. *HPG High* was determined to have a number average molecular weight of 1,412 g/mole and a polydispersity index of 1.16. This value is 3 times lower than the value obtained by GPC. Others have observed hyperbranched polymers with up to 5 times lower molecular weights obtained by MALDI MS as compared to GPC [21]. The number average molecular weight M_n_ calculated from the MALDI-TOF spectrum for *Control* is 1,164 g/mole which is in good agreement with the value of 1,253 obtained from GPC.

**Figure 6:**
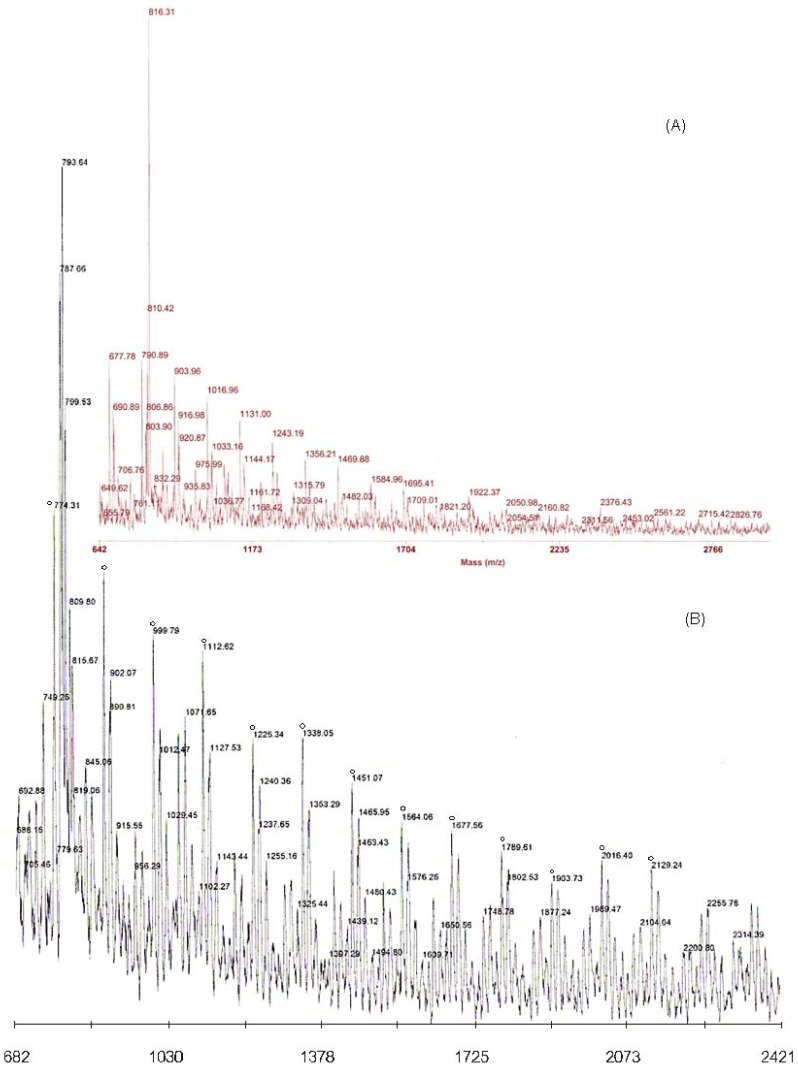
MALDI of copolymers (**A**) *HPG-High* and (**B**) *Control*.

Figure 6A shows the mass spectra of *Control* where the main peaks occur at m/z = 678; 791; 904; 1,019; 1,131; 1,243; 1,357; 1,470; 1,583; 1,895; 1,809; 1,922. The mass difference (m/z = 113) between these peaks precisely represents the molar mass of NIPAAm. Two additional subdistributions (starting with m/z = 690.83 and 693.95) are also observed with a mass unit difference of 113 between peaks in the respective series. Evidence of AAc (MW = 72) incorporation into the chains is provided by peaks occurring at *m/z* = 716.63 and 788.63; 790.84 and 862.79; 1,171.45 and 1,243.16. In each of these three series, the mass difference exactly corresponds to 72. The mass difference between peaks occurring at m/z = 1,243.16 and 1,372.64; 1,565 and 1,695.49; 1,738.77 and 1,869.05 precisely represents the molar mass of HEMA (*m/z* = 130). In addition, a peak mass difference of 318 corresponding to HEMALac is also observed (*m/z* = 716.63 and 1,035.05). No peaks were detected that corresponded to HPG in this control sample.

Figure 6B shows the MALDI MS of sample *HPG High*. Starting with the peak at *m/z* = 774.31, a series with mass difference corresponding to NIPAAm is observed (denoted by open circles above and to the left of peak mass numbers). A second series of peaks with mass unit difference of 130 is also observed in the spectra shifted to right of the first series by 15 mass units. Evidence of AAc incorporation into the chains is again observed. Peaks occurring at *m/z* = 999.79 and 1,071.65; 1,325.44 and 1,397.29; 2,129.24 and 2,200.80 all represent exact mass unit difference of 72. A peak mass difference of 130 corresponding to HEMA (*m/z* = 1,012.47 and 1,143.44) and 318 corresponding to HEMALac (*m/z* = 809.8 and 1,127.53) were also observed. We were mainly interested in detecting the incorporation of HPG-MA, and evidence of this was confirmed by the peak mass difference of 142 detected in three areas of the spectra: *m/z* = 774.31 and 916.31; 887.06 and 1,029.45; 1,338.05 and 1,480.43. One pair of peaks represented a mass difference of 74 (*m/z* = 1,802.53 and 1,877.24) which is exactly the mass for the monomer glycidol.

### 3.4. The LCST of Copolymers

**Figure 7:**
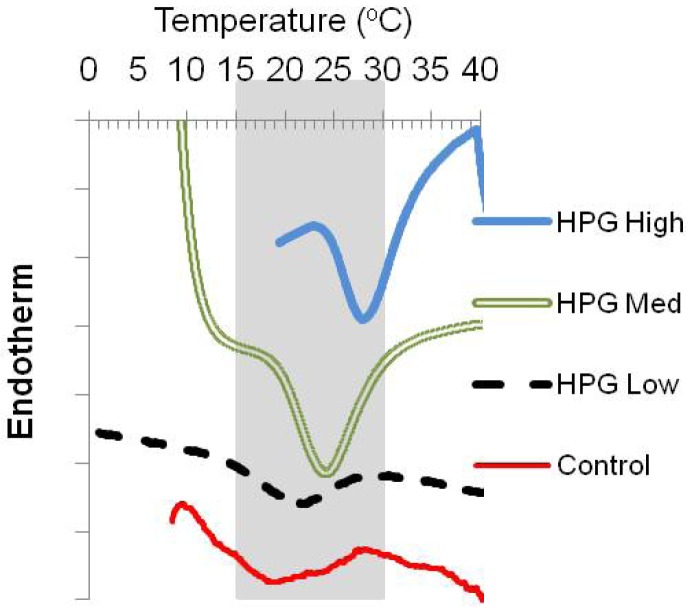
LCST determination by DSC analysis for all solutions of poly(NIPAAm-co-HEMAPLA-co-AAc-co-HPG-MA). The gray box focuses on the temperature range where all transitions were observed as indicated by the minimum in the endotherm trace.

The LCST of the different copolymers was determined based on abrupt changes in optical and thermal properties of the materials. DSC measurements of thermogelling solutions is a common method used to describe the phase transition temperature [9,13]. An endothermic peak occurs when a temperature is reached that induces hydrogen bond breaking in the water clusters around the hydrophobic domains and between the water molecules and amide bonds in the copolymers [30]. Typical DSC curves of copolymer solutions (16.7 wt% in PBS) showed broad but obvious endothermic peaks in the range of 20–28 °C as shown in Figure 7. The transition temperature shifts to lower values as the HPG content is reduced.

A similar phenomenon was observed from optical absorption data, where a jump in absorption is observed at a certain temperature. We did not perform light scattering studies of the copolymer solutions, however, others have observed a similar jump in optical absorption and attributed this to micelle formation at a certain temperature [13]. It should be noted that the transitions observed for the copolymers presented here are very close to room temperature and the instrument capabilities were limited to 25–45 °C. Therefore, the ramp up in the optical absorption was not observed over the entire critical range for all the copolymers. Instead, the peak maximum was the only observable part of the transition range for all but the *HPG High* sample.

**Figure 8:**
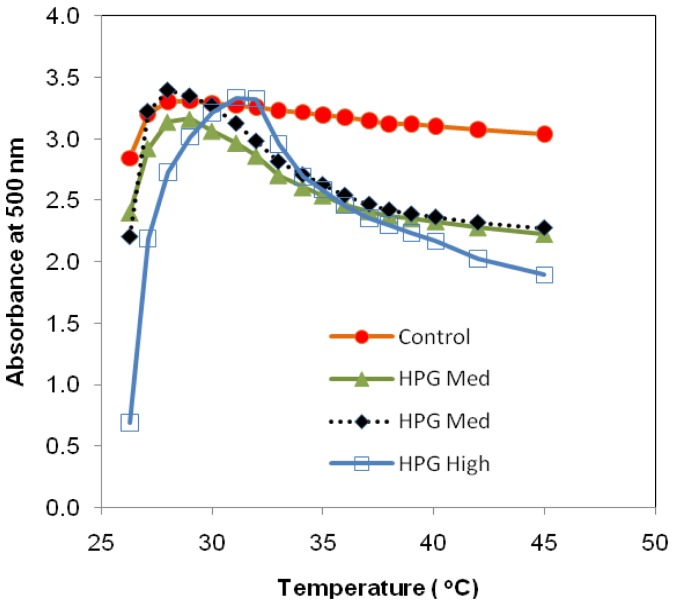
LCST determination by measurement of copolymer solution optical absorption as a function of temperature.

### 3.5. Cytotocity

**Figure 9:**
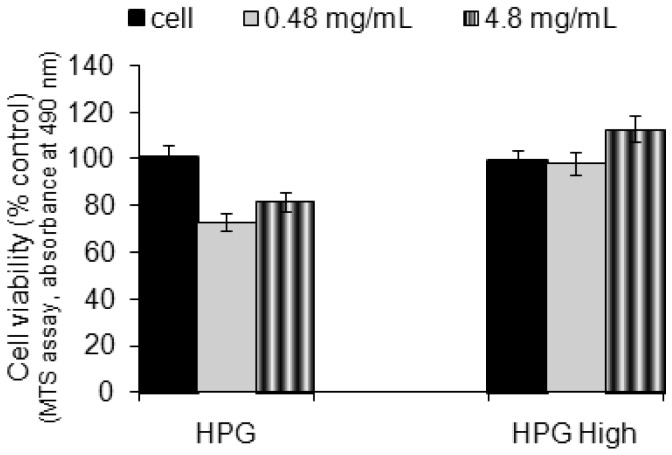
MTS assay to measure the cytotoxicity of HPG or copolymer *HPG High* at various concentrations. The materials were incubated with cultured fibroid cells for a total of 72 h before assessing cell viability in each group (n = 2). No statistically significant difference was noted relative to the control.

Uterine fibroid cells grown to 80% confluency show no negative effects on viability and metabolic activity after exposure for 72 h to medium containing either HPG or copolymer *HPG High* (Figure 9). This finding is promising for the potential application of the copolymer hydrogels as a localized drug delivery system for treatment of uterine fibroids.

### 3.6. Degradation Studies

**Figure 10:**
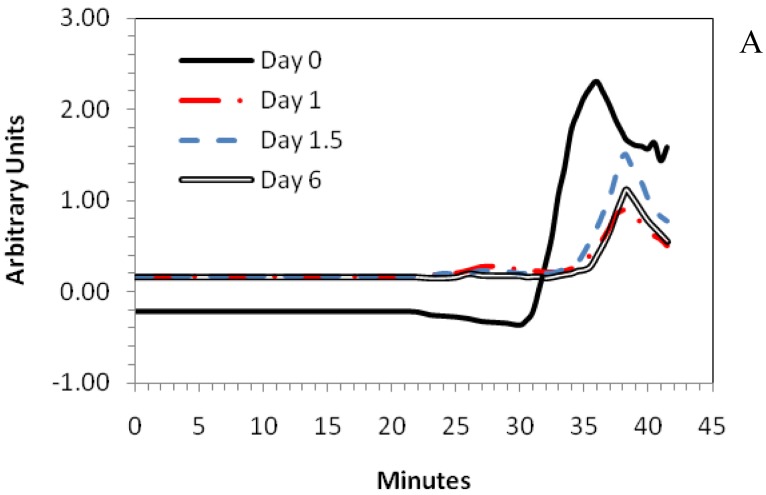

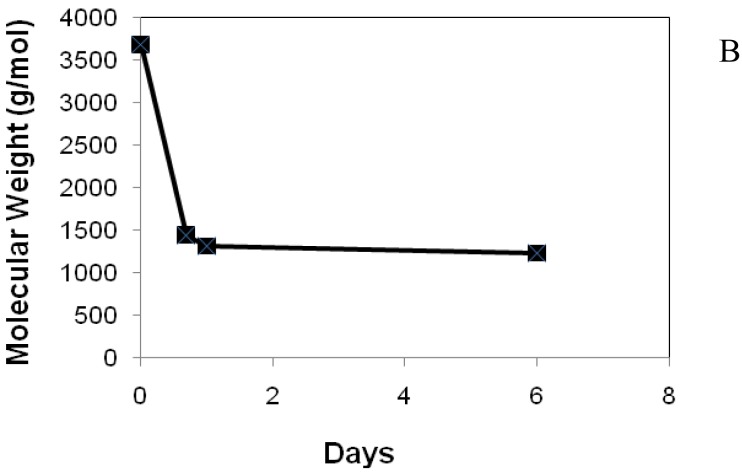
Degradation studies of 16.7 wt% copolymer gel *HPG High* at 37 °C showing GPC curves (A) and change in the molecular weight with time (B).

*HPG High* was characterized by GPC to determine modality and efficiency of the polymerization reaction [31] as well as the copolymer's degradation by a loss of molecular weight. The GPC chromatograms for *HPG High* lypholized samples became monomodal over successive days of degradation (Figure 10A,) and shifted towards lower molecular weights as degradation time increased. This result is consist with GPC curves for homopolymers of PLA (158.5 kg/mol) reported by Weir *et al.* to remain monomodal throughout successive weeks of degradation [32]. It should be noted that lyophilized samples of *HPG High* obtained after six days of incubation presented THF insoluble fractions even after stirring in THF for several hours. Presumably, the insoluble fractions represent the HPG component of the copolymer as HPG macromer is insoluble in THF. Thus, it is expected that the GPC traces should narrow in polydispersity as PLA chains are hydrolytically cleaved. In fact, the GPC chromatogram did show a decrease in polydispersity index (PDI) from 1.7 to 1.3 after complete hydrolysis. Before hydrolysis, the polydispersity is affected by both the composition and degree of polymerization. After hydrolysis, the affect of the composition on the polydispersity is reduced and the degree of polymerization becomes the main determinate of the polydispersity. In addition, although the GPC curve of *HPG High* before hydrolysis (Day 0, shown in Figure 10A) is broad, lower molecular weight impurities, unreacted starting materials, side products, and so forth, were not detected demonstrating the efficiency of the polymerization. There is no evidence of free HPG as this macromer is insoluble in the work-up solvents and HPG would only appear in the ESI and MALDI spectra (shown in Figure 2 and Figure 6 respectively) if it is covalently incorporated into the copolymer.

Figure 10B presents the GPC-generated findings that show a relatively fast decrease in molecular weight over a six day period. Hydrolysis of the LA-containing chains leads to mass loss. Within the first 16.5 hours, the copolymer has lost 95% of its molecular weight. These findings are consistent with reported PLA degradation kinetics which range from days to weeks based on crystallinity, molecular weight and distribution, orientation, unreacted monomer, and the presence of impurities [33].

**Figure 11:**
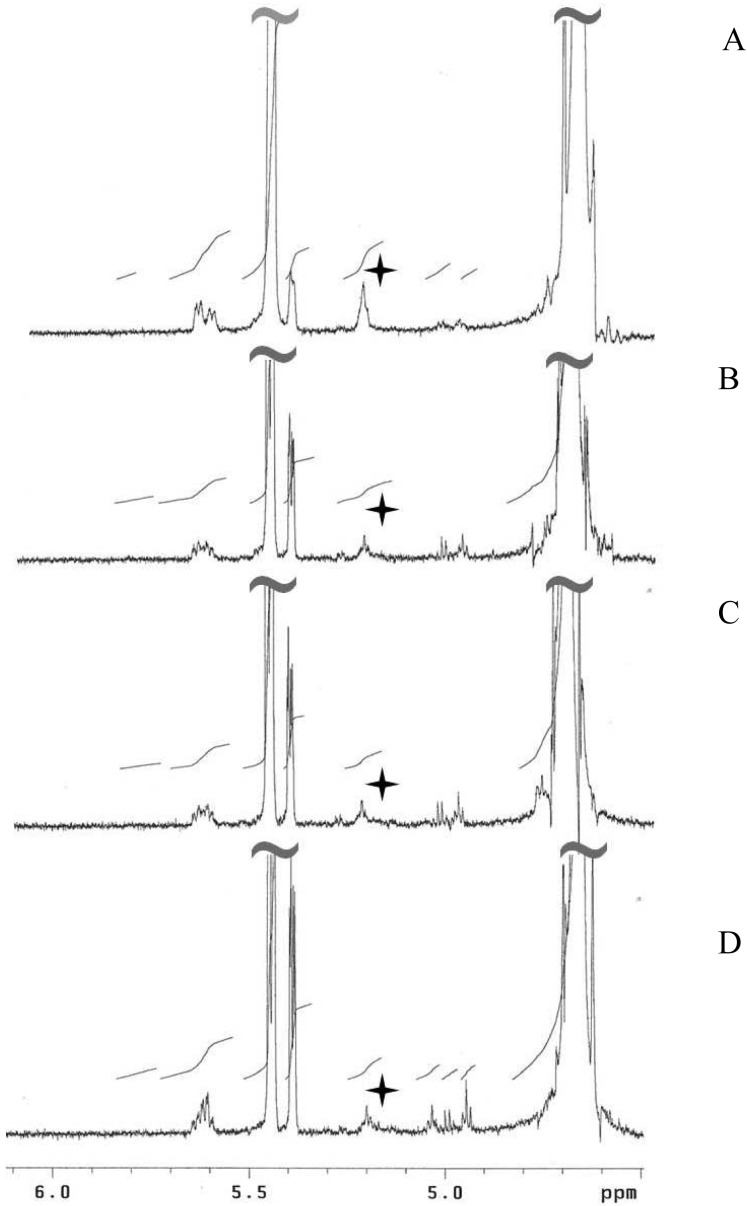
^1^H-NMR (D_2_O) spectral change during hydrolytic degradation of a representative poly(NIPAAm-co-HEMAPLA-co-AAc-co-HPG-MA) sample: (**A**) after 0 hours; (**B**) 15 hours; (**C**) 21 hours; and (**D**) 56 hours of degradation.

Figure 11 presents ^1^H NMR data showing spectral changes during the hydrolytic degradation of a representative poly(NIPAAm-co-HEMAPLA-co-AAc-co-HPG-MA) sample. In this figure, the HEMA-lactate peak (methane proton, 1H) at 5.2 ppm disappeared as the ester linkages of the polylactic acid spacers hydrolyzed during incubation. After 15 h, there is a sharp decrease in the peak and after 21 h the peak is no longer amendable to integration. These results are consistent with the finding reported from GPC analysis of the degradation product. This degradation trend is in agreement with a previous report on copolymers based on NIPAAm, HEMAPLA, and AAc [34]. Copolymer design alterations aimed at slowing the degradation rate would cause impacts on other copolymer properties such as the LCST.

## 4. Conclusions

Methacrylated HPGs have been incorporated into thermoresponsive hydrogels creating materials with added functional groups that could be easily manipulated in the design of future drug delivery systems. The copolymers were loaded with HPG up to 17% molar equivalents and displayed LCST as high as ∼30 °C for the highest HPG containing copolymer. All of the transition temperatures observed for the copolymers presented in this paper were below physiological temperature of 37 °C, and increasing the feed ratio of HPG beyond 17% would presumably further increase the sol-gel temperature. We are continuing to investigate the maximum HPG content required before negative effects are observed in the increasing LCST trend with the anticipation that functional, environmentally responsive materials for biological applications will be developed. The selected poly(NIPAAm-co-HEMAPLA-co-AAc-co-HPG-MA) (70/1/3.3/17) has attractive properties and is not toxic to cultured uterine fibroid cells. The novel copolymers presented herein represent a tunable thermoresponsive platform with potentially versatile functionality for drug delivery. Current ongoing research is focused on increasing the molecular weight of the copolymers, incorporating drugs into the thermogelling copolymers, studies of the effects of the chemical composition of the copolymers on the drug release kinetics, and evaluations of the *in vitro* and *in vivo* biosafety, bioefficacy, and bioavailability of the copolymer/drug systems.

## Figures and Tables

**Figure A1:**
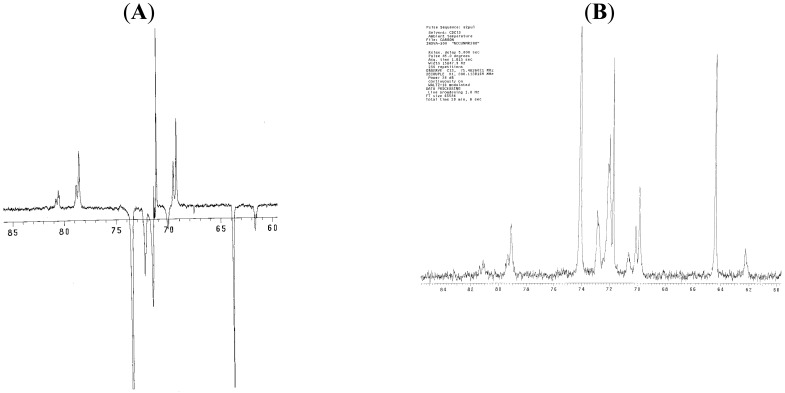
NMR spectra of hyperbranched polyglycerol (HPG-1 in Table S1) obtained in the anionic polymerization initiated with 1,1,1-tris(hydroxylmethyl)propane (TMP): (**A**) ^13^DEPT spectrum, (**B**)^13^C inverse gated spectrum.

**Figure A2:**
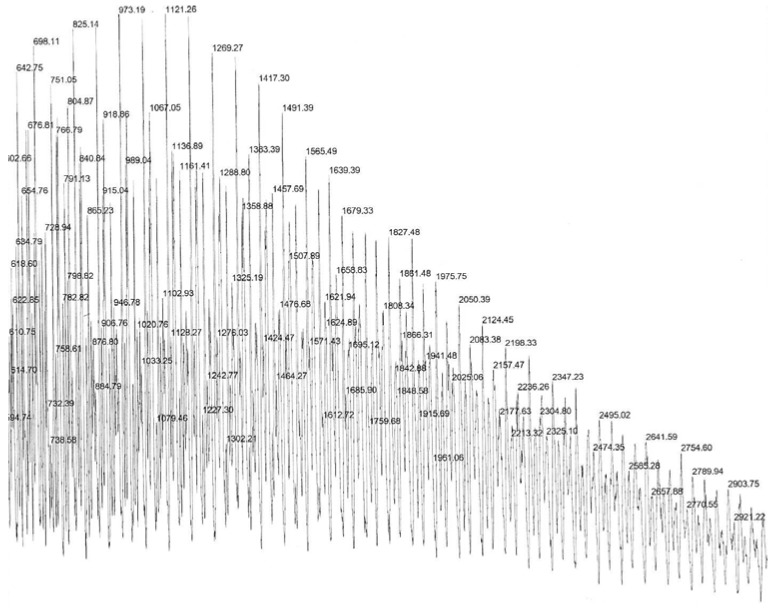
Representative MALDI-TOF mass spectra of HPG. Peaks are separated by 74 g/mole (the mass of the monomer glycidol).

## Appendix

**Table A1:** Interpretation of ^13^C NMR Inverse Gated Spectra of HPG.

**Region**	**Shift (ppm)**	**Relative Integral**		

		HPG-1	HPG-2	HPG-3
L_13_	81.0–82.0	1.00	1.00	1.00
D	79.5–80.5	2.10	2.15	1.90
2L_14_	73.5–74.5	6.94	6.69	2.58
2D,2T	72.0–73.5	11.18	11.17	8.18
L_13_, L_14_	70.5–72.0	3.88	3.96	2.25
T	64.0–65.0	4.05	3.86	0.59
L_13_	62.0–63.5	1.07	1.11	-*

**Structural Unit**	**Relative Abundance (%)**		
T	38	37	29
L_13_	9.4	10	4
L_14_	33	32	27
D	20	21	40

* No peak was observed.

**Table A2:** Results from ^13^C NMR and MALDI-TOF Mass Spectrometry.

**Sample**	**^13^C NMR**			**MALDI-TOF**	
	**DB**	**DP_n_**	**M_n_**	**M_n_**	***M_w_/M_n_***
HPG-1	0.48	16.3	1341	1367	1.15
HPG-2	0.50	18.1	1473	1257	1.18
HPG-3+	0.57	10.9	672	890	1.23

+ HPG-3 was prepared from S-glycidol. All other HPG samples were prepared from the racemic version of glycidol.
